# Variety in responses of wintering oystercatchers *Haematopus ostralegus* to near‐collapse of their prey in the Exe Estuary, UK

**DOI:** 10.1002/ece3.9526

**Published:** 2022-11-22

**Authors:** Joanne M. Morten, Ryan A. Burrell, Tim D. Frayling, Andrew N. Hoodless, William Thurston, Lucy A. Hawkes

**Affiliations:** ^1^ College of Life and Environmental Sciences University of Exeter, Hatherly Laboratories Exeter UK; ^2^ Game and Wildlife Conservation Trust Hampshire UK; ^3^ Devon & Cornwall Wader Ringing Group Ilfracombe UK; ^4^ Natural England, Sterling House, Dix's Field Exeter UK; ^5^ Met Office Exeter UK

**Keywords:** biologging, Eurasian oystercatcher, foraging behavior, *Haematopus ostralegus*, home range, shorebirds

## Abstract

Globally, habitat loss or degradation is a major threat to many species, and those with specific habitat requirements are particularly vulnerable. Many species of wading birds (Charadrii) are dependent upon intertidal sites to feed, but, as a result of anthropogenic pressures, the prey landscape has changed at many estuaries. Behavioral adaptations may be able to buffer these changes. In this study over multiple seasons, we aimed to investigate the foraging behaviors of wintering Eurasian oystercatchers in the Exe Estuary where mussel beds, the preferred prey at this site, have almost disappeared in the last decade. From 2018 to 2021, GPS tracking devices were deployed on 24 oystercatchers, and the foraging locations of adults, sub‐adults, and juveniles were determined. Of the 12 birds tracked over multiple winter periods, 10 used the same foraging home ranges but a juvenile and sub‐adult changed locations interannually. The dominant prey species at key foraging sites were assessed, and we found that younger birds were more likely to visit sites with lower quality prey, likely due to being at a competitive disadvantage, and also to explore sites further away. Individuals were generally consistent in the areas of the estuary used in early and late winter, and over 90% of locations were recorded in the protected area boundary, which covers the sand and mudflats of the Exe. These findings suggest high specificity of the current protected area for oystercatchers in the Exe Estuary, although, if the prey landscape continues to decline, younger individuals may provide the potential for adaptation by finding and foraging at additional sites. Continued monitoring of individual behavior within populations that are facing dramatic changes to their prey is essential to understand how they may adapt and to develop suitable management plans to conserve threatened species.

## Graphical abstract

For many waders reliant on intertidal mudflats, the prey landscape has changed, which may require behavioral adaptations for survival. GPS tracking devices were deployed on 24 wintering Eurasian oystercatchers in a declining population in the Exe Estuary, UK. Adults were consistent in their foraging locations both intra‐ and inter‐annually, whereas sub‐adults and juveniles showed greater flexibility and visited wider areas outside of the estuary. To buffer the loss of prey species in the Exe, oystercatchers may need to forage elsewhere, switch to less profitable prey, or increase foraging time.

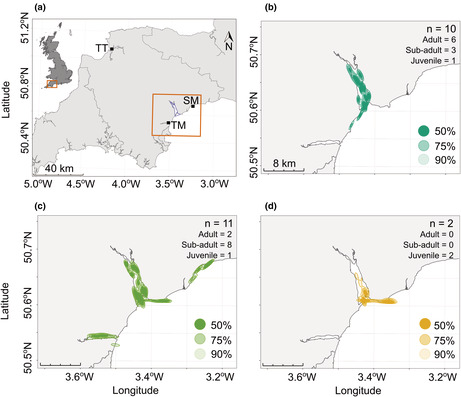

## 1 INTRODUCTION

During the Anthropocene, habitats have been lost, fragmented, or degraded across the globe at a dramatic rate, often more rapidly than species have been able to adapt (Brook et al., 2008; Otto, 2018). Synergistic interactions between habitat fragmentation and other threats such as climate change exacerbate species vulnerability, and species with smaller populations or ranges are at the greatest risk of extinction (Laurance & Useche, 2009; Pearson et al., 2014). Identifying a species' habitat preference and use is vital to guide appropriate conservation actions (Brambilla et al., 2009; Cañadas et al., 2005).

Wading birds (Charadrii), for example, often depend on tidal flats for foraging intertidal invertebrate prey, but more than 16% of tidal flats globally have been lost between 1984 and 2016, threatening many of the world's shorebird populations (Mu & Wilcove, 2020; Murray et al., 2019). Regime shifts are changes to species assemblages and abundance at regional or wider scales as a result of external factors (Kraberg et al., 2011). Within estuarine habitats, regime shifts of invertebrate prey can be caused by environmental or anthropogenic pressures such as warmer temperatures, extreme thermal events, and organic enrichment by, for example, sewage discharge, or commercial mariculture waste (Beukema et al., 2009; Pansch et al., 2018; Savage et al., 2002; Weston, 1990). Changes to the abundance or composition of estuarine benthic invertebrates will have knock‐on effects on their predators, for example impacting wader breeding success and winter survival (Bowgen et al., 2022). Wader species with a more specialized diet are more likely to be affected than generalist foragers that can compensate by switching to different prey, but changes to invertebrate prey will eventually affect all wading bird species (Bowgen et al., 2015).

With continuing advancements in biologging technology, animal behaviors can be explored with detail that could not be achieved using field observations alone (Bograd et al., 2010). Through biologging, insights into behaviors such as wintering site fidelity of migratory species and different migratory strategies within species have been revealed (Léandri‐Breton et al., 2021; van Bemmelen et al., 2019). Biologging has offered long‐term insights into the spatio‐temporal preferences of individual wild animals, foraging site fidelity, detailed behavior classifications, and also feeding specializations (Campioni et al., 2020; Shamoun‐Baranes et al., 2012; van der Kolk et al., 2020; Votier et al., 2017). Behavioral plasticity is a key response by species to anthropogenic change (Wong & Candolin, 2014), thus biologging allows us to study how species are adapting in space and time. Here we use GPS tracking devices to monitor the behavior of a declining population of Eurasian oystercatchers *Haematopus ostralegus* (hereafter oystercatchers), a near‐threatened species (BirdLife International, 2019), during their nonbreeding season.

Oystercatchers are a long‐lived, partially migratory wader that show high fidelity to their breeding and nonbreeding sites (van de Pol et al., 2014). Oystercatcher bill morphology reflects the variation in individuals' diets (Swennen et al., 1983). Some birds specialize as molluscivores, with chisel or hammer‐shaped bills, or worm feeders, with rounded and more pointed bills, whereas others have a more flexible, generalist diet (van de Pol et al., 2010). In the Netherlands, biologging studies have revealed that the time that wintering oystercatchers spend foraging each day is determined by their feeding strategy so that visually‐hunting worm specialists forage in tidal mud flats when exposed, and terrestrial habitats on bright nights, whereas shellfish specialists feed whenever the intertidal zone is sufficiently exposed (van de Pol et al., 2009; van der Kolk et al., 2020). Across the breeding and nonbreeding ranges of oystercatchers, there are multiple threats including habitat loss or damage by land reclamation, sea level rises, and agricultural intensification of field habitats, food shortages through bait‐digging, commercial shell‐fisheries, or encroachment and competition by invasive Pacific oyster *Crassostrea gigas*, and human disturbance by activities such as hunting, walking, and watersports (van de Pol et al., 2014). Prior work has modeled how oystercatcher populations may be affected by such external forcing factors including prey biomass requirements, disturbance, and commercial shellfishing (Durell et al., 2008; Goss‐Custard et al., 2019; Goss‐Custard, Durell, Sitters, & Swinfen, 1982; Stillman et al., 2010), and now, biologging allows detailed investigation of how individuals within a population are likely to be affected by environmental change, which can be used to further optimize such models.

The Exe Estuary, Devon, UK, (50.6°N, 3.4°W) hosts rich intertidal sand and mud flats of international importance. Extensive monitoring of the overwintering population of oystercatchers on the Exe Estuary began in the late 1970s, describing the foraging tactics, bill morphologies, age and sex structuring, competition, and disturbance of oystercatchers foraging on their dominant prey, blue mussels *Mytilus edulis* (Durell et al., 1993, 2001; Ens & Goss‐Custard, 1984; Goss‐Custard et al., 1981, 1983, 1984; Goss‐Custard, Durell, Sitters, & Swinfen, 1982; Goss‐Custard & Verboven, 1993), while alternative prey was consumed by less competitive juvenile birds (Goss‐Custard & Durell, 1983; Goss‐Custard, Durell, Sitters, & Swinfen, 1982; Goss‐Custard et al., 1981). Stillman et al. (2015) suggested that there were sufficient blue mussels in the Exe in 2013–2014 to support 1500–2000 wintering oystercatchers, but would have been unable to sustain a population as large as in the 1990s (*c*. 4500). Mussels were also commercially harvested across 31 mussel beds throughout the southern estuary, some of which were man‐made and maintained with imported spat (McGrorty et al., 1990). The mussel population of the Exe Estuary now appears to have completely collapsed in all but one surviving small mussel bed covering ~2500 m^2^, although this decline may have started as early as 1976 when there was evidence of natural spat failing to settle due to inclement weather reducing the availability of suitable habitats (Davies & Stephenson, 2017; McGrorty et al., 1990; Thomas, 2019). Furthermore, mussel spat tended to settle in existing mussel beds and was protected from predation by hiding within mature mussel byssus threads (McGrorty et al., 1990), so the recolonization will now be challenging with almost no beds for spat to settle to. Large cockles *Cerastoderma edule* are an alternative prey source for oystercatchers, but these have also apparently declined by approximately 70% in the Exe Estuary (Davies, 2017). The invasive Pacific oyster appears to have colonized the Exe as well, but incidences of oystercatchers handling and eating Pacific oysters are uncommon (Cadée, 2008; Davies & Stephenson, 2017; Herbert et al., 2018; Markert et al., 2013). Although there are likely to be several underlying causes for the decline of oystercatchers in the Exe Estuary, the potential changes to foraging areas and behaviors compared to those 40 years ago as a result of the regime shift of preferred prey species is a key factor to investigate. This study represents the first time that biologging has been used to record the individual movements of oystercatchers on the Exe Estuary, although the general movements of birds between mussel beds have been described in detail in previous work throughout the 1980s and 1990s (Goss‐Custard & Durell, 1983; Goss‐Custard & Verboven, 1993).

In this study, our aim was to use a combination of GPS tracking devices and field observations to study (i) home range sizes and use of the Special Protection Area (SPA) and surrounding areas (ii) movement patterns and the effects of extrinsic and intrinsic factors, (iii) the consistency and site fidelity of individual foraging sites, and (iv) prey intake and availability at the main foraging sites, and for all of these aims whether there are age‐related differences among individuals.

## 2 METHODS

### 2.1 Field site and study species

**FIGURE 1:**
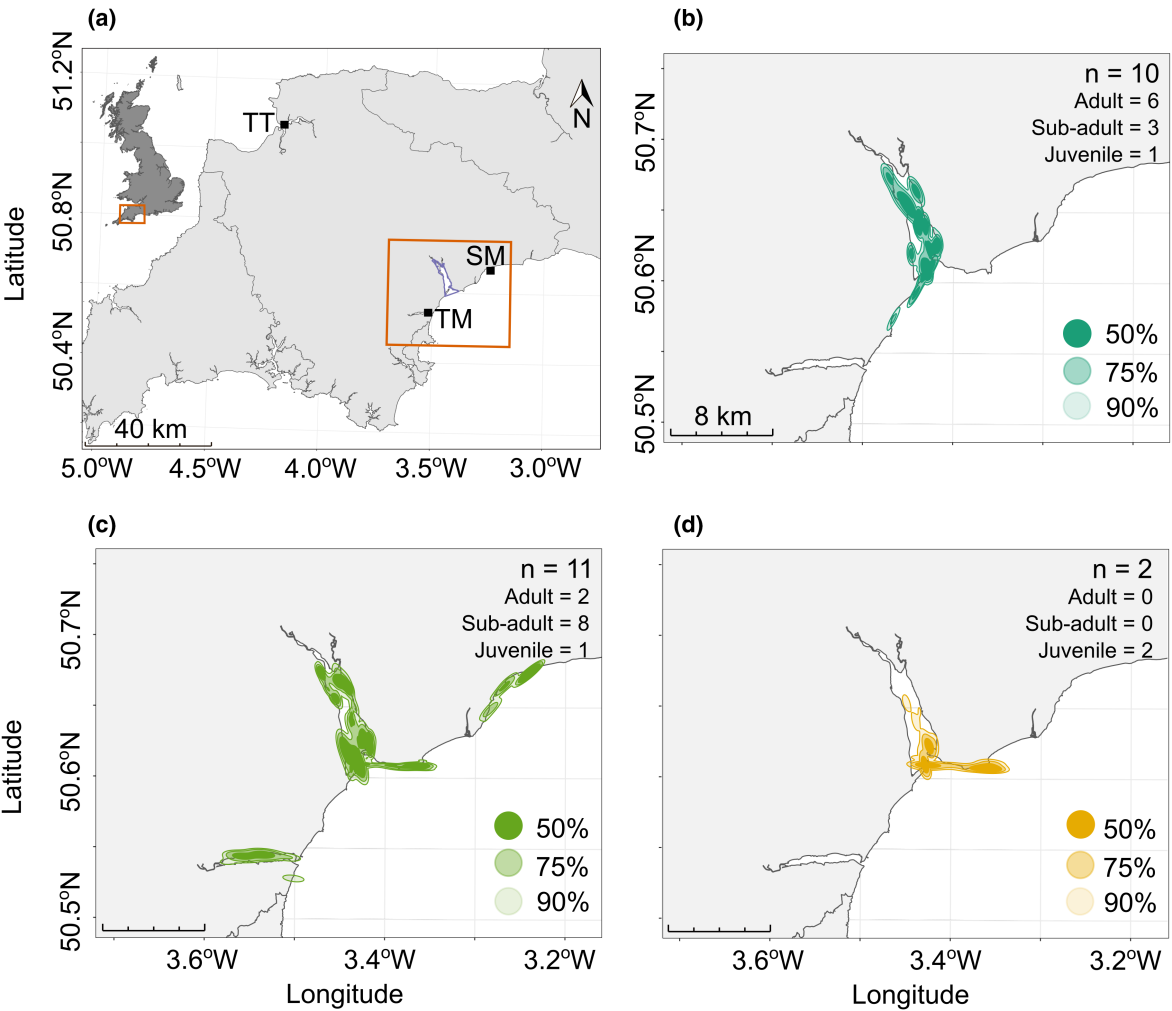
The study site location (a) and autocorrelated kernel density estimations (AKDEs) of oystercatchers tracked (b–d) during three overwinter periods (winter 1: November 2018 – March 2019, winter 2: August 2019 – March 2020, winter 3: August 2020 – March 2021). (a) The Exe Estuary Special Protection Area (SPA) is outlined within the orange box, and the locations of two neighboring sites (Teignmouth (TM) and Sidmouth (SM)), which were visited by the tracked oystercatchers, are labeled. A third site, the Taw Torridge (TT), where future oystercatcher tracking studies could include, is also labeled. The 50%, 75% and 90% AKDEs of the birds tracked during winter one (b), two (c), and three (d) are shown, with only the first winter of tracking data analyzed in cases where multiple years of data were collected for the same individual. The sample sizes indicate the total number of oystercatchers from which location data were used to estimate kernel size, and the distribution of age class is also shown.

The Exe Estuary, Devon, UK (Figure 1a) is an internationally important site and is a designated Special Protection Area (SPA), Site of Scientific Special Interest (SSSI), and a Ramsar site. It has an approximate area of 23.7 km^2^ that is 13 km long and 2.2 km at the widest point. It is used as an overwintering site by approximately 2125 oystercatchers every year (5‐year average Wetland Bird Survey (WeBS) data from 2015/16 to 2019/20; Frost et al. (2021)). The Exe Estuary is used predominantly as a wintering site by oystercatchers, although some nonbreeding individuals remain during the summer months. Sexually mature oystercatchers depart the Exe between February and March and breed throughout Western Europe (including Scotland, the Netherlands, and Norway, based on ring recoveries) before returning to the Exe between August and September to overwinter. The main oystercatcher roosting site is at the mouth of the Exe Estuary (Finger Point, 50.610°N, −3.427°W), where they aggregate at high tide. At low tide, the oystercatchers from this roost disperse to feed on estuarine mudflats and fields.

### 2.2 Animal capture

Between February 2018 and October 2020, 322 oystercatchers were caught and ringed by the Devon and Cornwall Wader Ringing Group at the main roost site, using either cannon nets or mist nets. All birds were ringed on the left tarsus with a unique metal ring from the British Trust for Ornithology (BTO). Of these 322 birds, 278 were fitted with color rings (a blue coded ring inscribed with a white two‐character alpha‐numeric code, and an un‐coded yellow ring above on the right tarsus), allowing individual identification in the field. Each oystercatcher was measured (head and bill length: from the back of the skull to bill tip, bill depth at the nostrils, bill length from the beginning of the feathering to the bill tip, bill tip width and depth) with Vernier calipers to 0.1 mm. Wing length was measured (1.0 mm accuracy) from the carpal joint to the tip of the longest primary. Mass was measured using a digital balance (to 1.0 g accuracy). Age was assessed using the bill and eye color, with plumage characteristics (Baker, 1974) and age categorized using EURING/BTO guidance (https://www.bto.org/sites/default/files/u17/downloads/about/resources/agecodes.pdf). Bill type was visually assessed as blunt, chiseller, or round bill (Swennen et al., 1983) by experienced wader ringers.

### 2.3 Tag deployment

Solar‐charged Global Positioning System (GPS) tracking devices (Pathtrack nanoFix GEO + RF) were deployed on 24 oystercatchers in November 2018 (*n* = 10), September – October 2019 (*n* = 11), and October 2020 (*n* = 3), and oystercatchers were released at the catch location, which is also the main roost site. Tags were attached to birds from three age classes: adult (hatched three or more years ago, exact age unknown; *n* = 8), sub‐adult (hatched before last calendar year, exact age unknown but not full adult; *n* = 11), and juvenile (hatched within the calendar year; *n* = 5). The tracking devices were attached using silastic leg‐loop harnesses (1.5 mm silicone cord passed through 3.0 mm OD silastic tubing; Polymax Ltd). Closed‐cell foam (3 mm height) was added to the base of each tag to reduce the chance of feather shading of the solar panel. Tag weight was approximately 7.5 g (model used in 2018) and 6.7 g (model used from 2019 onwards), and with an additional 2 g of attachment material, the total deployment weight was approximately 1.8% and 1.6%, respectively, of the oystercatchers' mean mass (515 ± 37.4 g). The tags recorded GPS locations at predetermined intervals and transmitted GPS data to a base station (that was positioned at the main roosting site) if the bird was within 300 m. Devices deployed in 2018 were scheduled to record hourly GPS locations and attempted to transmit data to the base station hourly between 1 November and 28/29 February. Between 1 March and 31 October, GPS locations were then recorded every 18 h, with remote download attempts every 21 days. The deployment schedule was revised in 2019 (due to excess battery drainage from hourly scheduling; Appendix 1), to record locations every 2 h between 04:00 and 22:00 from 1 August to 31 March, and attempt data transmission every hour. Between 1 April and 31 July, GPS locations were recorded every 12 h between 04:00 and 21:00 and download attempts were every 21 days. Tags were deployed by experienced and licensed individuals under a special methods permit issued by the BTO (ref 4173).

### 2.4 Tracking data analysis

Tracking data from the first set of deployments in 2018 (*n* = 10) were downsampled to match the temporal resolution of the tags deployed from 2019 onwards (i.e., 2‐hourly resolution with an off period from 22:00 to 04:00). The home range area estimations were calculated for the full and downsampled data in 2018 and visually compared (Appendices 2 and 3). Following a visual assessment of the tracking data, any locations recorded more than 20 km from the roosting site were excluded as these were part of migration. Not all of the tracked oystercatchers migrated, and any locations recorded outside of the “winter” period (from departure for migration or when the sampling reduced on 1 March, whichever occurred first) until the average return date of tracked oystercatchers (14th August), were excluded. The minimum distance that an individual could have traveled between successive locations was calculated as the great circle distance between the two locations using the R package *geosphere* and expressed as the distance traveled per hour (i.e., for two‐hourly intervals, the distance was divided by two). The 25%, 50%, 75%, 90%, and 95% home ranges were estimated for each individual using autocorrelated kernel density estimation (AKDE) in the continuous‐time movement modeling framework (R package ctmm; Calabrese et al., 2016). This method accounts for the autocorrelation inherent in tracking data and also copes with irregular sampling intervals (Mitchell et al., 2019). AKDEs for each age class and each winter period were spatially summed in R using the *sf* package (Pebesma, 2018). The overlap between the 50%, 75%, and 90% kernels and the boundary of the Special Protected Area of the Exe Estuary were calculated using the *sf* package in R. All spatial calculations were performed on projected data (ESPG: 27700).

### 2.5 Environmental data

Daily sunrise, sunset, and twilight times for the study site were determined using the R package *suncalc* (Thieurmel & Elmarhraoui, 2019). Tidal data (high and low tide times and heights) were extracted from Polpred (https://noc.ac.uk/business/marine‐data‐products/offshore) for Exmouth Approaches (the nearest buoy to the study site), and tidal state was appended to locations as the nearest 1 h before or after high and low tide. Weather conditions were assessed, and the number of days below freezing and with strong winds determined whether winters were mild or severe. Hourly local air temperature (to the nearest 1°C) and wind vector (speed and direction, to 0.1 m s^−1^ accuracy) data were accessed and downloaded from the nearest weather station at Exeter airport, which is approximately 13.6 km from the roost location at the mouth of the estuary (Integrated Surface Dataset (Global), National Centers for Environmental Information, National Oceanic and Atmospheric Administration (NOAA) https://www.ncei.noaa.gov/access/search/data‐search/global‐hourly). Wind speed data were derived from equipment recording to the nearest whole knot. To reflect this level of accuracy in the recording equipment, the wind speeds were converted back to knots and rounded to the nearest whole number.

### 2.6 Foraging behavior

From January 2019 to March 2019, and October 2019 to February 2020, eight key and accessible foraging sites in and around the Exe Estuary were identified from the tracking data. During winter 1 (2018/19) and winter 2 (2019/20), these sites were visited to record foraging behavior, and 323 oystercatchers were observed foraging for 5 min (*n* = 111 and *n* = 212, respectively). Each estuarine site was usually visited at least 1.5 h before low tide, and visits to the terrestrial sites varied from 2 h before high tide to 3 h before low tide. Color‐ringed oystercatchers were selected as focal individuals for observation to prevent resampling of the same individual. In cases where no ringed oystercatchers were present, unringed birds were followed. Focal oystercatchers were watched foraging for a minimum of 300 seconds using a Swarovski ATX 30‐70x95 scope. The type of prey consumed (mussel, cockle, peppery furrow shell *Scrobicularia plana*, winkle *Littorina* sp., Pacific oyster, estuarine worm *Hediste divericolor*, earthworm *Lumbricus* sp., or leatherjacket *Tipula* sp. larvae) was recorded, with its size estimated in relation to the bill length of the bird. The total number of prey consumed in 5 min was calculated, as well as the time between the prey item being found and consumed or abandoned, which was recorded as handling time. A Mann–Whitney *U* test compared whether the number of prey consumed in 5 min in field and estuarine habitats differed. Where marked individuals were observed on multiple occasions at the same site, only the first observation per bird was included in analyses. Unringed birds that were followed were assumed to be different individuals on different days (there are *c*. 1800 unringed oystercatchers in the Exe Estuary), but it is possible, though unlikely, that the same individuals were resighted and recounted. To test the effect of including these birds, we removed all but the unringed individual observations on the first day per site and compared them with the whole dataset using a two‐sample test of proportions without continuity correction. At 5 of the 8 sites, the prey proportions were not significantly different. At the other three sites some, but not all, of the prey types were significantly different (Appendix 4). Since the prey composition did not alter at most sites, or if it did not for all prey types, we accepted the small chance of pseudoreplication and included all unringed birds in the analyses. The prey quality at each site was estimated using published values (Wroot, 1985; Zwarts & Wanink, 1993) to assess the relative profitability of each of the areas used by oystercatchers in the Exe Estuary.

### 2.7 Prey abundance

To estimate the prey available to oystercatchers in different parts of the estuary and the surrounding fields, prey samples were collected from six of the eight key foraging sites as identified from the tracking data (two sites were inaccessible or landowner permission could not be obtained). Sample areas within key sites were pseudo‐randomly selected, and sampled in January – March 2019, and January – February 2020, during the period where prey biomass is likely to be at its smallest (Honkoop & Beukema, 1997). Logistical constraints prevented sampling outside of these periods. A total of 18 quadrat samples were collected at estuarine sites and nine at field sites. A 0.5 m × 0.5 m quadrat was cast randomly at each selected sample area and the ground where it landed was excavated to a depth of approximately 10 cm. The maximum bill length of oystercatchers caught on the Exe Estuary since February 2018 was 9.35 cm and a 10 cm excavation should have been deep enough to encompass all prey available to a foraging oystercatcher. To collect all the macroscopic prey at estuarine sites the contents were sieved, collected, weighed (wet mass), and identified to the lowest taxonomic level possible. The length, width, and height of the shells were measured using Vernier calipers (to the nearest 0.1 mm), and the wet mass of the flesh was measured to the nearest 0.01 g using a Pesola PPS200 balance. Prey items collected in fields were identified to genus and the overall wet mass per quadrat was calculated. Prey masses were converted to estimated ash‐free dry weight and approximated calorific values were calculated using published conversion factors and values (for details see Appendix 5).

### 2.8 Statistical analyses

To avoid pseudoreplication, only locations recorded in the first year of deployment were analyzed for any individuals with multiple winters of data, unless otherwise stated.

Prey availability likely deteriorates over the winter, with decreases in both abundance and the mass of bivalves, which is expected to lead to an increase in the times that oystercatchers spend feeding (Goss‐Custard, 1977; Goss‐Custard et al., 1996). To investigate whether this occurred, we compared the mean distances traveled by oystercatchers every 2 h during early (1 October – 31 December) and late (1 January – 31 March/ departure from the Exe) winter with paired *t*‐tests (only including birds with locations during both periods, *n* = 20).

To test whether oystercatchers moved at specific times of the tidal cycle, the tidal state (time to or from the closest low tide), when oystercatchers moved more than 1 km in 2 h was converted into radians using the *astroFns* R package (Harris, 2012), and then into a circular data object using the *circular* R package (Agostinelli & Lund, 2017). The times (or tidal states) were then tested for uniformity with the Hermans‐Rasson test (using the R package CircMLE; Fitak & Johnsen, 2017) owing to multimodal peaks in activity (Landler et al., 2018, 2020).

One‐way ANOVAs with post hoc Tukey tests compared whether there were differences in the mass, head, and bill length and wing length of the three age classes caught from February 2018 – October 2020. One might expect that less dominant oystercatchers, such as females, which typically have longer bills (Durell, 2007), or juveniles, which typically have shorter wings (Zwarts et al., 1996), would be less able to forage in areas with higher quality prey, or greater prey abundance. To test whether the distances that oystercatchers traveled (with lower quality foraging sites further from the roost) related to dominance, individual size was used as a proxy. Heavier oystercatchers also consume larger prey more rapidly (Goss‐Custard et al., 2006). As such they may not need to visit multiple sites to meet their daily requirements. Individual measures (mass, head and bill length, and wing length) were correlated with the average distance they traveled every 2 h using a Pearson's correlation (all variables except mass were normally distributed (Shapiro–Wilk test *p* > .05), which was transformed into a negative reciprocal of mass (which was normally distributed).

To understand inter‐seasonal consistency in behavior, the 75% kernel areas were calculated in the R package *sf* for the 12 individuals with data collected for two or more winters. The size of the area (km^2^) in which the first and second winter kernels intersected was also calculated in the *sf* package. The overlap of an individuals' first and second winter 75% kernels was calculated as the intersection area divided by the first winter kernel area and multiplied by 100 to give a percentage value. To determine whether tracking year could explain any differences in home range areas, generalized linear mixed models (R package *lmer*) with and without tracking year as a fixed effect (oystercatcher ID was a random effect) were compared with a likelihood ratio test. The 75% kernel areas of the first and second winter data were then compared using paired *t*‐tests.

Unless otherwise stated all analyses were conducted in R (version 3.5.1).

## 3 RESULTS

Tracking devices recorded and transmitted location data for a median of 80 days (2018 tags with hourly schedule, *n* = 10) and 471 days (2019 and 2020 tags with 2 hourly schedule and off overnight, *n* = 14), with battery voltage a limiting factor and some variation possibly caused by differential shading of the solar panels on the devices (Appendix 1). One tracking device deployed in October 2020 only functioned for 8.4 days, so was excluded from all analyses. Considering all data downsampled to the coarsest sampling resolution (2 h and off from 22:00 to 04:00) a total of 49,924 locations were recorded from all birds tracked in this study. Only 12 devices were transmitted for more than one winter period. A total of 14,758, 32,018, and 3148 locations were recorded for birds tagged in 2018 (total locations for downsampled data with same temporal resolution), 2019, and 2020, respectively.

### 3.1 Use of the Exe Estuary SPA and wider areas

**FIGURE 2:**
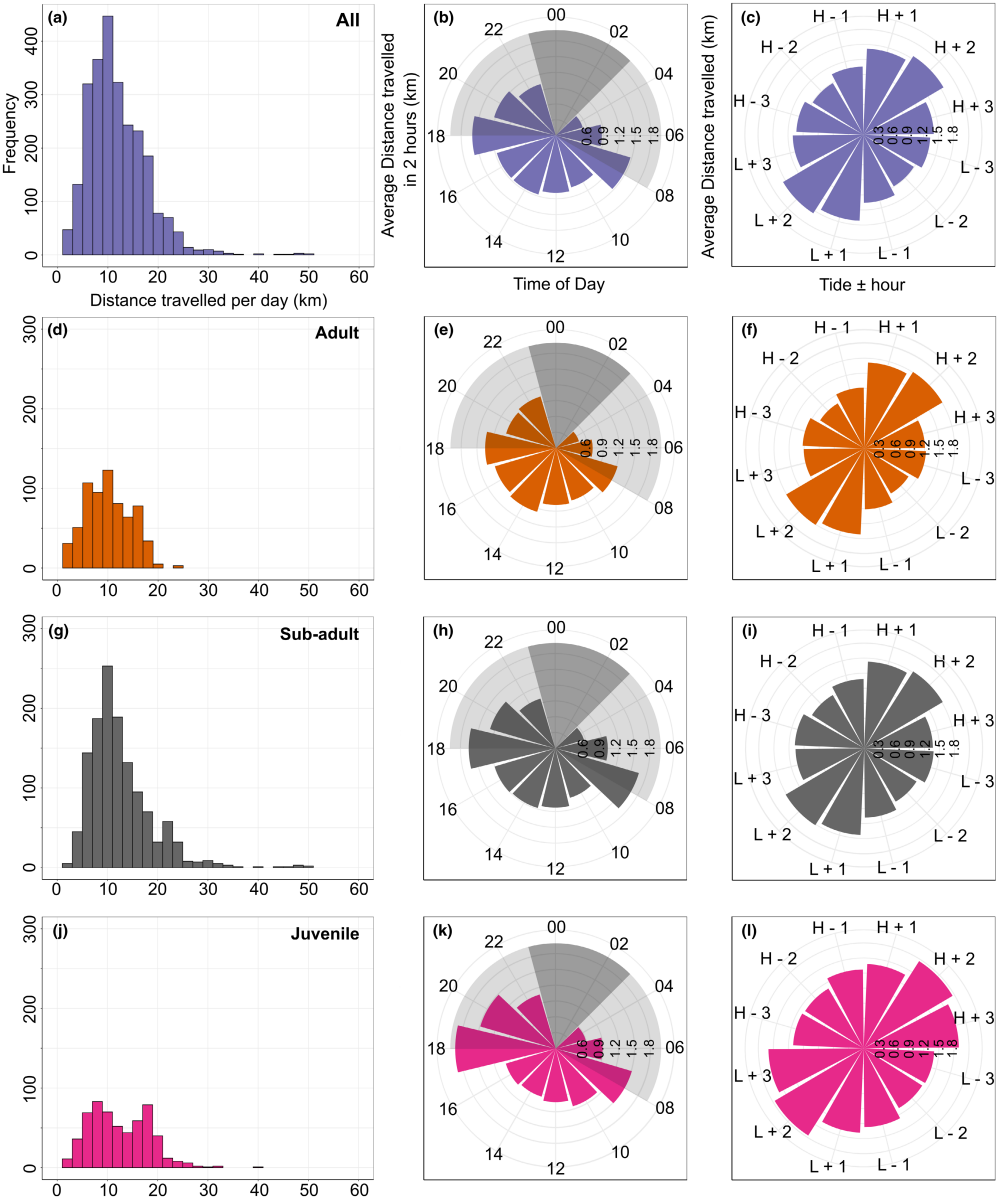
The daily activity of oystercatchers tracked during three winter periods (2018–2021) in the Exe Estuary, UK. The distances traveled per day, per 2‐h time period, and throughout the tidal cycle are shown for all tracked individuals (*n* = 23; a–c), and also for adults, sub‐adults, and juveniles only (*n* = 8, d–f; *n* = 11, g–I; *n* = 4, j–l, respectively). (a, d, g, j) histograms show the mean total distance traveled per day. (b, e, h, k) circular bar plots of the mean distances traveled during two‐hourly time windows. The pale grey polygons indicate the period between sunset and sunrise, and dark grey polygons indicate when tracking devices were switched off to conserve battery power. (c, f, I, l) The mean distances traveled 1–3 h before high (“H ‐“) and low (“L ‐“) tides and after high (“H +”) and low (“L +”) tides.

Oystercatchers traveled a median distance of 10.40 km (0–50.3 km) per day (Figure 2a), with foraging sites within the Exe Estuary varying from 0.7 to 7 km from the main roosting site. The three age classes traveled similar median distances per day (adult: 9.90 km, range 0.17–23.37 km; sub‐adult: 11.1 km, range 0.16–50.29 km; juvenile 9.27 km, range 0.002–39.70 km; Figure 2d,g,j), but small sample sizes prevented statistical analysis. Tagged oystercatchers generally remained within the SPA boundary (90.2% of locations were from within the SPA area) throughout the winter period. Their usage of the SPA did not alter during the winter (90.2% of locations within the SPA during the early winter: October – December, and 92.6% in the late winter: January – March). There was no significant difference in the distance traveled every 2 h in early and late winter (mean 1.0 and 1.02 km per 2 h, respectively, paired *t*‐test *t*_19_ = −0.37, *p* = .72). Five oystercatchers (1 adult, 3 sub‐adults, and 1 juvenile) visited sites outside the Exe: the Teign Estuary (southwest of the Exe), Dawlish (west of the mouth of the Exe), Sandy Bay (east of the mouth of the Exe), and Sidmouth (northeast of the Exe; Figure 1).

In winter 1 (2018/19) tracked oystercatchers generally remained within the Exe Estuary and 97.3% of recorded locations were within the SPA boundary. More adults were tracked in winter 1 (*n* = 6), than sub‐adults (*n* = 3) or juveniles (*n* = 1). In winter 2 (2019/20) the 11 oystercatchers captured at the start visited areas outside of the Exe Estuary and 85.6% of locations were within the SPA boundary. In winter 2 there were more sub‐adults tracked (*n* = 8) than adults (*n* = 2) or juveniles (*n* = 1). In winter 3 (2020/21) the three tracked oystercatchers (all juveniles), visited locations outside of the Exe Estuary more than in previous years, and only 70.4% of locations were in the SPA boundary. The merged home ranges were largest in winter 2 (50% kernel estimation = 11.2 km^2^, 90% = 32.8 km^2^, *n* = 11; Figure 1c), compared with winter 1 (50% = 6.9 km^2^, 90% = 18.4 km^2^, *n* = 10; Figure 1b) or winter 3 (50% = 2.8 km^2^, 90% = 11.8 km^2^, *n* = 2; Figure 1d). The weather conditions in all 3 years were relatively mild; from 14 August until 1 March, the number of days below 0°C was 28, 13, and 17 in winter 1, 2, and 3, respectively, and wind gusts only reached “near gale” strength three times (Appendix 6).

The combined core area (50% home range) of tagged oystercatchers covered 14.9 km^2^, which overlapped by 66.9% with the SPA. Of the 23 tracked birds (excluding the device that worked for <9 days), the core area of 19 birds overlapped with the SPA more than 80% (Table 1). The remaining four oystercatchers (three sub‐adults and one juvenile) had core home ranges that overlapped with the SPA less than 60%, and all foraged in areas outside of the Exe Estuary. Adult birds used smaller home ranges than both sub‐adults and juveniles (merged 90% home ranges: 12.0, 32.6, and 19.2 km^2^, respectively; Appendix 7). The core 50% home areas were also smallest in adult birds compared with sub‐adults and juveniles (4.4, 11.1, and 5.7 km^2^, respectively). Adult home ranges overlapped with the SPA boundary to a greater extent than either sub‐adult or juvenile home ranges (percentage overlap with SPA boundary of adult 50% and 90% kernel estimations: 88.9% and 73.4%, sub‐adult: 63% and 50.4%, and juvenile: 75.3% and 73.3%). All eight tracked adult birds foraged in the south of the estuary, although one did additionally forage outside of the SPA, whereas all individuals that visited the north of the estuary were either sub‐adults or juveniles. No juveniles had home ranges exclusively in the south of the estuary, although six sub‐adults did (Table 1).

**TABLE 1:** The median distances, home ranges (HR) used, and their location within the Exe Estuary, and the overlap of the home ranges with the special protected area (SPA) for 24 oystercatchers tracked during the winter months from 2018/19 to 2020/21.

Oystercatcher identity	Age class	Year deployed	Body mass (g)	Median distance traveled per day (IQR) (km)	75% (50%) home range (km^2^)	Percentage overlap of 90% (75%, 50%) HR with SPA	Key region of estuary
2L	Adult	2018	670	5.7 (3.5)	3.81 (1.81)	86.5 (88.4, 90.6)	South
J3	Adult	2018	604	2.7 (2.7)	1.01 (0.50)	94.3 (97.4, 98.9)	South
5H	Adult	2018	563	5.5 (3.1)	4.00 (1.84)	91.8 (96.8, 100)	South
5N	Adult	2018	522	4.8 (3.6)	2.68 (1.27)	98.4 (99.8, 100)	South
8Y	Adult	2018	490	4.7 (4.2)	2.54 (1.06)	65.8 (74.4, 90.5)	South and outside of estuary
Y5	Adult	2018	470	4.0 (4.3)	3.42 (1.67)	96.6 (98.0, 100)	South
3K	Sub‐Adult	2018	494	3.5 (2.6)	1.96 (0.86)	97.7 (98.6, 100)	South
8E	Sub‐Adult	2018	488	4.3 (2.7)	2.63 (1.23)	89.0 (93.2, 98.5)	South
8T	Sub‐Adult	2018	544	5.4 (2.9)	1.98 (1.02)	99.0 (99.2, 99.5)	South
6M	Juvenile	2018	484	2.0 (2.7)	5.18 (2.27)	87.1 (89.3, 93.1)	North
N2	Adult	2019	490	9.9 (4.8)	3.41 (1.56)	86.8 (86.9, 85.5)	South
6A	Adult	2019	546	3.0 (3.0)	0.80 (0.33)	76.2 (85.2, 98.1)	South
M0	Sub‐Adult	2019	490	7.3 (4.0)	3.16 (1.55)	91.0 (90.7, 90.6)	North
K1	Sub‐Adult	2019	495	11.7 (3.9)	2.44 (1.17)	52.2 (44.7, 30.3)	Outside of estuary
2K	Sub‐Adult	2019	542	10.4 (4.9)	2.43 (1.11)	99.6 (100, 100)	South
L2	Sub‐Adult	2019	583	7.3 (4.0)	3.44 (1.61)	92.4 (96.1, 98.8)	South
A5	Sub‐Adult	2019	526	5.7 (4.0)	7.58 (3.27)	82.7 (84.6, 85.5)	Both north and south
E6	Sub‐Adult	2019	499	9.2 (3.9)	5.53 (2.47)	47.1 (51.4, 57.3)	Outside of estuary
J7	Sub‐Adult	2019	522	9.4 (8.7)	4.70 (2.35)	93.5 (95.2, 97.8)	South
C9	Sub‐Adult	2019	540	7.6 (6.3)	7.63 (3.59)	45.8 (51.1, 52.9)	North and outside of estuary
AJ	Juvenile	2019	539	6.0 (9.3)	2.78 (1.26)	90.0 (92.5, 98.5)	Both north and south
AM	Juvenile	2020	407	11.8 (2.3)	3.85 (1.80)	47.0 (43.2, 34.3)	Outside of estuary
CE	Juvenile	2020	503	1.5 (7.0)	3.06 (1.21)	94.4 (92.7, 95.5)	Both north and south
CL	Juvenile	2020	577	13.4 (7.9)	7.61 (4.18)	92.0 (95.7, 97.2)	Both north and south

During the summer months, nonbreeding oystercatchers that remained within the Exe Estuary generally foraged in the same locations as during the winter months (Appendix 8). However, due to the lower temporal sampling resolution from April to August, only a visual assessment to compare area usage was possible. In the absence of breeding adults, three of the eight individuals that remained in the Exe appeared to only visit sites in the south of the estuary, and not the northerly sites that were also visited during the winter months. It is, however, possible that they still visited the northern sites during the summer, but locations were not captured due to the sampling resolution.

### 3.2 Timing of movements

Oystercatchers exhibited crepuscular behavior with the largest mean distances traveled at dawn and dusk (mean ± SD at dawn: 1.07 ± 1.67 km, dusk: 1.13 ± 1.54 km, day: 0.92 ± 1.50 km, night: 0.88 ± 1.34 km; Figure 2b). All three age classes exhibited this pattern of movement (Figure 2e,h,k). Oystercatcher movements were also linked to the tidal cycle, such that tagged oystercatchers traveled the greatest distances one and 2h after high and low tides (Figure 2c; Hermans‐Rasson T: 8230, *p* = .002). Both adults and sub‐adults traveled greater distances 1–2 h after each tide (Figure 2f,i), but juveniles traveled greater distances an hour later, 2–3 h after high and low tides (Figure 2l). After each high tide, the tagged oystercatchers traveled from the high tide roost to a foraging site. They reduced their movement rates in the hours preceding low tide when they were foraging in exposed estuarine habitats. One to three hours after low tide (during the flooding tide), the oystercatchers traveled greater distances again, likely returning to roosting sites, where they remained until the following receding tide.

### 3.3 Individual variation

**FIGURE 3:**
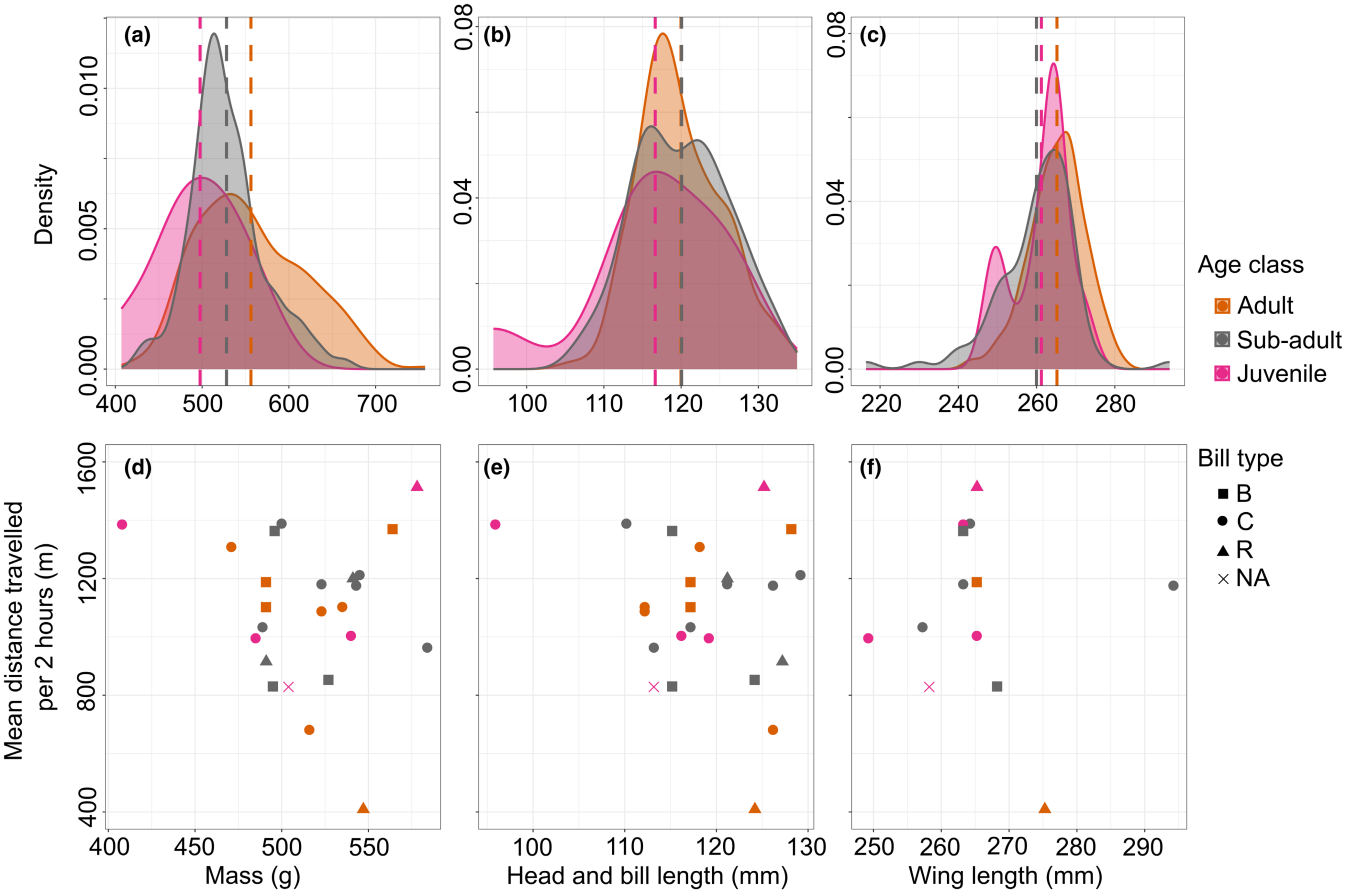
Density plots of the (a) mass, (b) head and bill length, and (c) wing length of all oystercatchers captured between February 2018 and October 2020. The different age classes are shown by the different colors (adult = orange, sub‐adult = grey, and juvenile = pink). Dashed lines show the mean biometric value of captured oystercatchers in these age classes. A subset of 24 oystercatchers were GPS tracked and the relationship between the (d) mass, (e) head and bill length, and (f) wing length and the mean distance traveled over a 2‐h period during the winter months (14th August until 1st March) are plotted. The three different bill types (B = blunt, C = chiseled, R = rounded) are represented by different shapes.

Adult oystercatchers caught from February 2018 to October 2020 (*n* = 322) were significantly heavier than sub‐adults or juveniles, and also had significantly longer wings than sub‐adults, but there was no significant difference in the head and bill length between the age classes (Figure 3a–c; Appendix 9). Biometric measurements (mass, head and bill length, and wing length, recorded at capture) were not related to movements (distances traveled per 2 h, Figure 3d–f).

**FIGURE 4:**
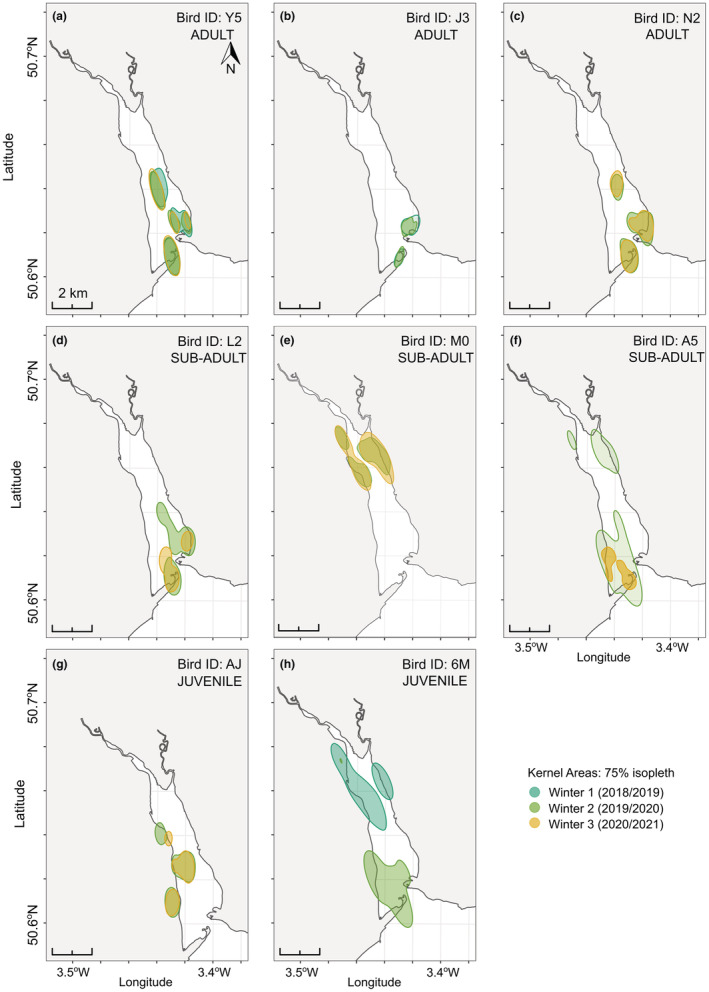
The 75% autocorrelated kernel density estimations (AKDE) for eight (of twelve) oystercatchers with location data collected for two or more winters.

Twelve birds transmitted data for two or more winter periods. The foraging sites of ten birds did not vary inter‐seasonally (Figure 4 and Appendix 10). The 75% estimated kernel areas were generally consistent inter‐seasonally, with a large amount of overlap. Variation in the size of the home ranges could not be explained by either the tracking year (i.e., the first or second year each bird was tracked) or the calendar year (when weather conditions might have varied; *p* = .38). There was also no significant difference in the size of the first and second winter home ranges (*t* = −0.624, df = 11, *p* = .55), and for eight individuals there was more than a 70% overlap between the two winter home ranges (Table 2). The two birds that changed foraging sites from the first to second winter were a juvenile and sub‐adult during the first winter. They both moved from lower‐quality areas in the north of the estuary (see 3.4 Foraging behavior and prey availability) to higher‐quality areas in the south in winter 2 (Figure 4f,h).

**TABLE 2:** The 75% AKDE areas of oystercatchers tracked for more than one winter period, and the overlap area and percentage overlap between the two winter kernel estimates

Oystercatcher identity	Age class	75% AKDE area (km^2^)		Intersection area (km^2^)	Overlap between intersection area and winter 1 area (%)
		Winter 1	Winter 2		
5H	Adult	4.00	3.81	3.33	83.2
6A	Adult	0.80	0.46	0.40	50.4
J3	Adult	1.01	0.81	0.75	74.3
N2	Adult	3.41	2.98	2.80	82.1
Y5	Adult	3.42	2.73	2.51	73.4
2K	Sub‐Adult	2.43	24.93	2.43	100
A5	Sub‐Adult	7.58	1.49	1.49	19.6
C9	Sub‐Adult	7.63	9.06	5.96	78.1
L2	Sub‐Adult	3.44	1.84	1.38	40.0
M0	Sub‐Adult	3.16	4.31	3.03	95.9
6M	Juvenile	5.18	5.28	0.01	0.2
AJ	Juvenile	2.78	2.17	1.95	70.4

### 3.4 Foraging behavior and prey availability

**FIGURE 5:**
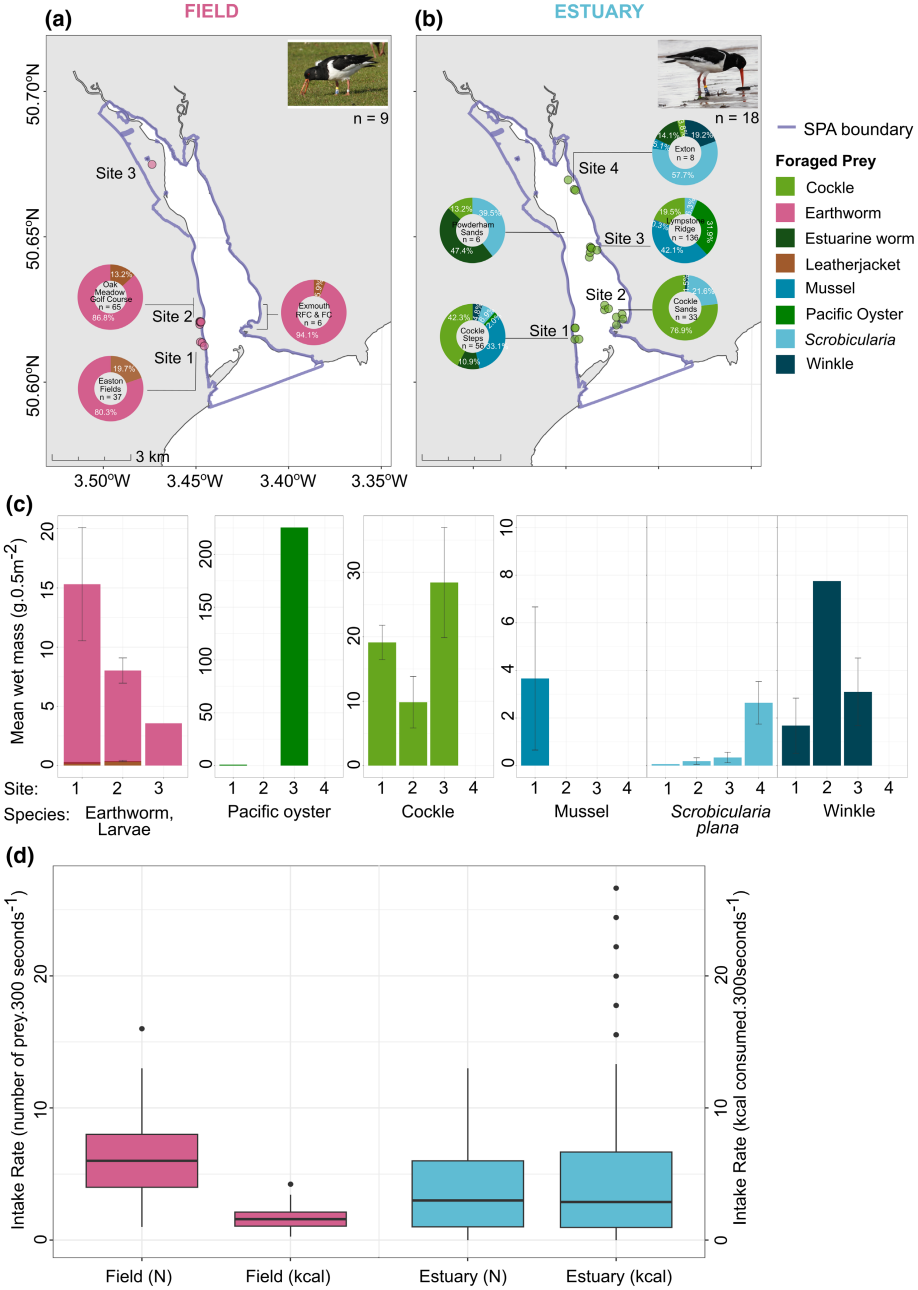
Oystercatcher foraging behavior and the composition and distribution of their prey in the Exe Estuary, UK. Foraging behaviors were observed at field (a, *n* = 3) and estuarine (b, *n* = 5) sites. Pies show the proportion of each prey type consumed at each site, and the total number of foraging observations per site is shown at the centre of each pie. Prey samples were collected at key field and estuarine sites (pink, *n* = 9, (a) and green, *n* = 18, (b) circles, respectively), which were selected by field observations and tracking locations. (c) The mean wet mass of prey species per 0.5 m^2^ quadrat sampled at each site with standard error bars. (d) Boxplots compare the number of prey consumed (N) and the approximated calorie content (kcal) in the field and estuary habitats during 5‐min foraging observations.

There were 212 foraging observations within the Exe Estuary SPA and a total of 112 in surrounding field locations. Of these, 239 included uniquely ringed individuals on the first observation at a site, and the remaining were observations of unringed birds. The numbers of prey items consumed in 5 min varied with habitat type (Figure 5d). Prey was consumed at a significantly faster rate in field locations (median = 6 prey items every 5 min, IQR = 4) than in estuarine locations (median = 3 prey items every 5 min, IQR = 5; Mann–Whitney *U* test: *W* = 6490, *p* < .001). At field locations oystercatchers predominately consumed earthworms (Lumbricidae *sp*.; Figure 5a), whereas, in the estuary, the prey species consumed varied by site (Figure 5b), and with the prey distribution in the estuary. At sites in the south, oystercatchers predominately foraged on cockles and mussels, whereas at sites in the north the dominant prey was *S. plana* or estuarine worms (Figure 5b). Although the energy content of prey items is dynamic and varies seasonally (Goss‐Custard, 1977), a very approximate estimation of the energy content of earthworms, mussels, cockles, *S. plana* can be cautiously made (Appendix 5), and suggests that estuarine prey likely are more valuable than earthworms from field locations (Figure 5d). Of the estuarine prey species foraged by oystercatchers, mussels probably contain the most calories per item, followed by cockles, and then *S. plana*. Differences in energy content indicate that within the Exe Estuary, sites in the south are more profitable areas to forage compared with sites in the north of the estuary.

Prey species found in the estuarine quadrat samples included cockles, *S. plana*, Pacific oysters, and winkles, and in the field quadrat samples both terrestrial earthworms and leatherjacket (*Tipula* sp.) larvae were collected. The number of estuarine prey items varied from 4 to 121 per quadrat, and 0.1 to 4.5 g of wet mass per quadrat. The number of terrestrial prey items varied from 25 to 82 per quadrat, and 0.13 to 0.38 g of wet mass per quadrat. However, prey type varied by location, such that terrestrial sample locations were dominated by earthworms (95.8%), the three southern estuary sites by cockles (Site 1 = 75.6%, Site 2 = 86.6%, Site 3 = 80.1%), and 100% by *S. plana* in the northern site (Figure 5c). In total only six mussels were sampled in the 18 estuary quadrats, and all were at one site in the south of the estuary.

## 4 DISCUSSION

### 4.1 Use of the Exe Estuary SPA and wider areas

The Exe Estuary is a highly protected site with Ramsar, Special Protection Area, and Site of Special Scientific Interest (SSSI) designations. The GPS tracking data presented here revealed that over 90% of transmitted locations from the 24 oystercatchers were within the protected area, but despite this, the population of oystercatchers wintering in the Exe has declined by almost two‐thirds (Frost et al., 2020). The population decline is unlikely to be uniform across age groups, because in general, younger birds used larger home ranges, and were more likely to explore areas away from the Exe Estuary, than adults, which remained in relatively restricted foraging sites. There is a subset of the population that roost and forage in terrestrial habitats outside of the protected area, and it is therefore possible that some behaviors of overwintering Exe Estuary oystercatchers were omitted in the tracking data, which should be addressed in future work. There was little difference in the distances traveled in early and late winter, or inter‐seasonally, indicating that oystercatchers use similar foraging and roosting areas throughout the overwintering period in the Exe, and individuals likely forage in areas that they are familiar with in different years.

Oystercatchers not only need to consume sufficient prey to survive (their “Physiological Requirement,” PR), but the availability of prey in the environment (their “Ecological Requirement,” ER) probably needs to be up to eight times larger to maintain PR by locating enough suitably sized and easy to handle prey, particularly with competition from other birds (Goss‐Custard et al., 2019, 2004; Stillman et al., 2016). Recent modeling suggests that neither disturbance from people nor mussel harvesting should significantly impact the wintering population of oystercatchers in the Exe (Goss‐Custard et al., 2019). The question then remains whether oystercatchers are managing to meet their PR following the collapse of mussels (Henly, 2021b; Thomas, 2019) and whether food limitation may therefore be one driver of the observed population decline. Oystercatchers have been well documented to primarily feed on mussels in the Exe Estuary throughout the 1980s and 1990s (Cayford & Goss‐Custard, 1990; Goss‐Custard et al., 1981, 1993; Goss‐Custard, Durell, McGrorty, & Reading, 1982), so there is an increased imperative to understand how the change in prey might impact the population.

In the Burry inlet, south Wales UK, where cockle prey stocks crashed between 2004 and 2010, overwintering oystercatchers moved to nearby Carmarthen Bay to buffer the loss of prey (Bowgen et al., 2022). It appears that the tracked birds from the Exe Estuary may be seeking alternative foraging sites to meet their daily energy requirements too. Of the 24 tracked oystercatchers, five were recorded regularly foraging away from the Exe Estuary, either along the coast or in the neighboring Teign Estuary. A lack of historic records, however, means that it is possible that foraging outside the Exe is not a new strategy, and may have occurred in a subset of the population prior to the declines of overwintering oystercatchers and their prey. Additionally, the buffering effect of the Teign Estuary may be limited as from 2012 to 2018 the population of mussels there has also declined, which has led to the temporary closure of mussel, oyster, clam, and periwinkle harvesting (Devon and Severn IFCA, 2019; Stephenson & Clark, 2018). By contrast, the estimated tonnage of mussels increased from 2020 to 2021 in the Taw Torridge Estuary (Henly, 2021a), which is located approximately 70 km northwest of the Exe (Figure 1a). Given the importance of connected habitats to buffer regime shifts in prey, future studies should broaden their scope across multiple foraging sites to better unravel the complicated processes driving oystercatcher population declines (Bakker et al., 2021), and biologging will be a critical tool for documenting the movement of wintering birds between sites. Eventually, if prey abundance is too low in the Exe Estuary to support sufficient oystercatchers and other shorebirds, remedial action may need to be taken. Prior to the cessation of mussel fishing in the 1950/60s in the Exe, many of the beds were artificially created, but the beds are no longer maintained using traditional methods (McGrorty et al., 1990; Stillman et al., 2015). Restoration of mussel beds, however, is a complex process with low success rates or failures (e.g., de Paoli et al., 2015; Schotanus et al., 2020). Increasing the area of mussel lays may also have minimal effects due to short exposure times (see Stillman et al., 2015).

### 4.2 Timings of movements

Oystercatcher movements are strongly related to the tidal cycle, which is just under 25 h in the Exe Estuary, and intertidal prey availability is likely to be overwhelmingly important for them (Schwemmer & Garthe, 2011). However, in the present study, tracking data highlighted crepuscular peaks in activity, which were unexpected because oystercatchers are known to forage during darkness (Goss‐Custard & Durell, 1987; Hulscher, 1976; Shamoun‐Baranes et al., 2012; Sutherland, 1982). They therefore did not always appear to need to return to roost, as the timing of low tide would have varied throughout the winter. Daytime foraging is probably still more efficient regardless of an oystercatcher's prey specialization, and the time taken to locate prey increases at night (Schwemmer & Garthe, 2011; van der Kolk et al., 2020). Oystercatchers could therefore have waited until sunrise to move to foraging locations and returned to the roosting site at sunset regardless of when the lowest point in the tidal cycle was. Generally, crepuscular behavior is unusual in waders, although it has been reported in dunlin *Calidris alpina* and Western sandpipers *C. mauri* (Ruiz et al., 1989).

### 4.3 Foraging behavior and prey availability

It appears that despite the loss of important mussel beds, the southern parts of the Exe Estuary provide more profitable prey (such as cockles) than the northern estuary (dominated by *Scrobicularia plana*). There will also be seasonal differences in the energy content of each region because during the summer spawning season the energy content of female bivalves laden with eggs increases, whereas males decrease (Zwarts & Wanink, 1993). However, the dominant prey types would likely remain the same. Oystercatchers could respond to the changes that have occurred in prey in the Exe by switching their diet, or by changing their foraging method. Oystercatchers' bills change shape with their diet as a result of their method used to open bivalves, or by probing worms (Swennen et al., 1983; van de Pol et al., 2009). Handling efficiency is, however, reduced for at least a few weeks while their bill morphology adapts (Hulscher, 1984; Wanink & Zwarts, 1996). This could mean that rapid changes in prey, such as the loss of mussel beds during severe storms in 2014 (Davies & Stephenson, 2017), could lead to starvation. However, Edwards et al. (2021) showed that oystercatchers foraging on limpets *Patella* spp. responded to short‐term changes in environmental conditions and maximized efficiency by altering their foraging technique.

Interspecific competition with other shorebirds for estuary prey may also be important to consider. Specialized foragers such as Eurasian curlew *Numenius arquata* are less likely to show dietary flexibility (although they can feed on less profitable terrestrial worms; Bowgen et al., 2015). They may compete more strongly with oystercatchers for limited prey than more opportunistic foragers such as sanderlings *Calidris alba* that spend more time foraging in sites with lower food availability and intake rates (Lourenço et al., 2015). The invasion of Pacific oysters, which has transformed some Exe Estuary mussel beds into oyster reefs, may have changed the prey landscape by creating new habitats and supporting crustaceans and estuarine worms, which in turn can be predated by curlew and oystercatchers, although reefs tend to be avoided by other shorebirds such as godwits *Limosa* sp. (Herbert et al., 2018; Markert et al., 2013). Oystercatchers are considered generalist foragers, eating a variety of prey species and sizes, and biologging approaches such as accelerometry could reveal changes in their foraging strategies (Bakker et al., 2021; Shamoun‐Baranes et al., 2012; van der Kolk et al., 2020).

### 4.4 Individual variation

Tracking data highlighted strong site fidelity and inter‐seasonal consistency in foraging locations and all five adult birds that were tracked for two or more seasons foraged in the same home ranges every year. The only birds that changed their foraging sites were one sub‐adult (of five with two or more years of data) and one juvenile (of two with two or more years of data). In the Dutch Wadden Sea, Verhulst et al. (2004) reported that oystercatchers did not redistribute to areas with higher cockle density, indicating high foraging site fidelity and low plasticity. The population decline in the Exe is unlikely to be equally distributed across the age classes and should be explored further. Younger oystercatchers are generally subordinate to adults (Goss‐Custard & Durell, 1983). Differences in dominance could explain the propensity of sub‐adults and juveniles to forage in areas outside of the Exe; as they mature and become more competitive they could forage more successfully with the richer sites in the south of the Exe Estuary. There may also be sex‐based differences since female oystercatchers are more likely to be worm‐feeding mudflat feeders with pointed bills, whereas males are more likely to be mussel‐feeding hammerers (Durell et al., 1993). Historically, due to feeding on lower profitability prey, females were the more vulnerable sex (Durell et al., 1993). However, with the deterioration of mussel beds, male oystercatchers may also have lowered intake rates. Overall, though, in a landscape of shifting and diminishing prey, adult birds may fail to adapt, while younger birds may offer the greater possibility for the adaptive capacity of the population. However, higher juvenile mortality rates may limit their influence on population demographics (Goss‐Custard et al., 1982b). A lower juvenile survival rate is likely to be common among many more overwintering oystercatcher populations and is of conservation concern given that prey decline or collapse is not unique to the Exe (e.g., Burdon et al., 2014).

In this study, we focused on the area usage and foraging behaviors of oystercatchers following dramatic regime shifts to their foraging landscape. There is, however, a suite of other factors that might impact this overwintering population, as well as declining oystercatcher populations elsewhere. Oystercatchers and other wader species are likely to be negatively impacted by climate change, with sea level rises further exacerbating habitat losses at both breeding and nonbreeding sites (Galbraith et al., 2002). Although oystercatchers were tracked during mild winters, it is possible that during very harsh winters, they could exhibit behavioral shifts, such as prolonging intertidal foraging time when field habitats are frozen and terrestrial prey are inaccessible. Increasing incidences of extreme weather, such as winter storms or heat waves, could impact species indirectly, for example by affecting their foraging ability, or via direct mortality (Clark, 2009; Sutherland et al., 2012). In the UK, some form of anthropogenic pollution, which could include sewage run‐off, chemical pollution, or agricultural pollution, affects all rivers (Environmental Audit Committee, 2022). Pollution could further contribute to prey regime shifts, and some prey species, such as *S. plana* or gastropods, are more sensitive to pollution from sewage discharge or fertilization run‐off than others (Cabral‐Oliveira et al., 2014; Gonçalves et al., 2016). Some species may be able to adapt behaviors to these rapid changes, whereas others may be more vulnerable meaning conservation actions are highly complex. A holistic and collaborative approach is necessary to conserve migratory species, such as oystercatchers, as these animals rely on multiple sites during the breeding and nonbreeding seasons, and often also use rich stop‐over sites en route (Runge et al., 2014). Monitoring the detailed behaviors of species of conservation concern is essential to determine how they respond to wide‐ranging threats and whether any adaptive changes are rapid enough (Sih et al., 2011).

## 5 CONCLUSIONS

We have used GPS tracking devices to highlight how oystercatchers may respond to changes in the composition and abundance of prey species at an individual level throughout a winter period. Long‐term monitoring over months and multiple winters have offered valuable insight into individual intra‐ and inter‐seasonal consistencies in foraging sites, likely to influence the conservation measures required for the population as a whole. Individuals sought alternative foraging sites outside of the estuary and consumed prey not previously recorded within the Exe (Pacific oysters), likely in response to declines in prey abundance. In contrast to mature birds, younger individuals had larger home ranges and were more likely to explore areas outside of the Exe. Therefore, more naïve oystercatchers may be more likely to successfully find alternative foraging sites elsewhere, if the invertebrate prey landscape continues to alter, although this will come at an energetic cost of traveling further. Within the wider county area, there may be alternative estuaries to buffer prey regime shifts, but similar declines in mussels and cockles are also occurring in the neighboring estuary (Stephenson & Clark, 2018), and the adults in particular remain faithful to the Exe Estuary site. It is likely that in response to continuing losses of their preferred prey species, oystercatchers will need to meet their daily energy requirements by foraging outside of the Exe, switch to less profitable prey, or spend longer periods foraging per day.

## AUTHOR CONTRIBUTIONS

**Joanne M. Morten:** Conceptualization (equal); data curation (equal); formal analysis (lead); methodology (equal); writing – original draft (lead); writing – review and editing (equal). **Ryan A. Burrell:** Conceptualization (equal); data curation (equal); methodology (equal); writing – review and editing (equal). **Tim D. Frayling:** Conceptualization (equal); funding acquisition (lead); supervision (equal); writing – review and editing (equal). **Andrew N. Hoodless:** Conceptualization (equal); methodology (equal); writing – review and editing (equal). **William Thurston:** Resources (equal); supervision (supporting); writing – review and editing (equal). **Lucy A. Hawkes:** Conceptualization (equal); formal analysis (supporting); methodology (equal); supervision (lead); writing – original draft (supporting); writing – review and editing (equal).

## ACKNOWLEDGEMENTS

We are grateful to all the DCWRG members who helped with the catches, and in particular Pete Potts, Robin Ward, and Lizzie Grayshon who led the cannon‐net catches. We thank Teignbridge District Council, Devon Wildlife Trust, Warren Golf Course and Starcross Golf for permission to access their land, and Teignbridge Ranger Service and Habitat Mitigation Officers for their help on catch days. Gary Brodin provided valuable advice on scheduling of the GPS tags. Thanks to Lee Collins, Ivan Lakin, Kevin Rylands and all those who have provided details of re‐sighted birds. We are grateful to John Goss‐Custard for his expertise and assistance during field work and for informative discussions, and to Richard Stillman for his advice on monitoring foraging behavior. We thank Matthew Witt for support with tidal data. We appreciate the advice of Humphrey Sitters and Roger and Barbara Swinfen, who ran DCWRG up to the early 2000s, on re‐starting studies on Exe oystercatchers. We also thank the editors and reviewers for their valuable comments and contributions.

## Data Availability

Data openly available in a public repository that does not issue DOIs: https://www.movebank.org/cms/movebank‐main, reference number: 649979264.

## APPENDIX 1

The change in the battery levels of solar‐charging GPS tracking devices deployed on oystercatchers (*n* = 24, listed by unique leg ring in panel, right) since November 2018. The two colors indicate differing sampling resolution (orange “winter” periods = hourly resolution and on 24 h, blue “winter” periods = 2 hourly resolution and off from 22:00 to 04:00). The bars at the bottom indicate “winter” periods, with lower sampling resolutions outside these ranges (orange “summer” sampling resolution = 18 h, blue “summer” sampling resolutions = 12 h). Horizontal dashed line shows a battery voltage of 3.7 V, below which tags are unable to continue to record and send GPS data.

## APPENDIX 2

The home range kernel estimations (50%, 75%, and 90%) of 10 oystercatchers tracked during the winter of 2018/19 at an hourly sampling resolution for 24 h per day (left columns, red) compared with the same data downsampled to match the 2‐hourly sampling resolution for 18 hours a day (right columns, blue).

## APPENDIX 3

The home range estimations of 10 oystercatchers tracked during the winter of 2018/19 at an hourly sampling resolution and 24 h per day compared with the estimations on the same locations but at a lower sampling resolution of one location per 2 h and off from 22:00 and 04:00

**Table:**

Bird ID	Kernel estimation	Home range area (km^2^)	
		Full sample resolution (hourly and no off period)	Downsampled resolution (2 hourly and off period)
2L	50	1.61	1.8
2L	75	3.47	3.81
2L	90	5.51	6.08
3K	50	0.67	0.86
3K	75	1.58	1.96
3K	90	2.9	3.63
5H	50	1.55	1.84
5H	75	3.37	4.00
5H	90	5.48	6.28
5N	50	1.11	1.27
5N	75	2.36	2.68
5N	90	4.07	4.42
6 M	50	1.84	2.27
6 M	75	4.40	5.18
6 M	90	8.30	9.36
8E	50	1.16	1.24
8E	75	2.37	2.63
8E	90	3.96	4.39
8T	50	0.89	1.02
8T	75	1.80	1.98
8T	90	2.90	3.05
8Y	50	0.85	1.06
8Y	75	2.13	2.54
8Y	90	3.94	4.57
J3	50	0.42	0.50
J3	75	0.85	1.01
J3	90	1.49	1.72
Y5	50	1.97	1.67
Y5	75	3.95	3.15
Y5	90	6.16	5.29

## APPENDIX 4

A comparison of the proportion of prey type eaten by foraging, unringed oystercatchers on the first day only (known to be different individuals) and on all observation days (individuality cannot be certain). A statistically significant 2 sample test of proportions means that the first day only prey proportions are different from the prey proportions for all observations.

**Table:**

Site	Prey	Proportion of prey foraged by unringed birds: First‐day observations only (%)	Proportion of prey foraged by unringed birds: All observations (%)	2 sample test of proportions without continuity correction
Oak Meadow Golf Course	Earthworm	87.0	86.8	*χ* ^2^ = 0.011 *p* = .9165
	Leatherjacket	13.0	13.2	*χ* ^2^ = 0.011 *p* = .9165
Eastdon Fields	Earthworm	76.3	80.3	*χ* ^2^ = 0.879 *p* = .3484
	Leatherjacket	23.7	19.7	*χ* ^2^ = 0.879 *p* = .3484
Lympstone	Cockle***	46.8	19.5	*χ* ^2^ = 57.4 *p* < .001
	Estuarine worm	1.1	0.3	*χ* ^2^ = 1.77 *p* = .1834
	Mussel	43.1	42.1	*χ* ^2^ = 0.057 *p* = .8122
	Oyster***	4.8	31.9	*χ* ^2^ = 55.86 *p* < .001
	*S. plana*	4.3	6.3	*χ* ^2^ = 1.089 *p* = .2966
Cockle Sands	Cockle	74.0	76.9	*χ* ^2^ = 0.42 *p* = .5169
	*S. plana*	24.9	21.6	*χ* ^2^ = 0.549 *p* = .4587
	Winkle	1.2	1.5	*χ* ^2^ = 0.086 *p* = .769
Cockle Steps	Cockle	36.6	42.3	*χ* ^2^ = 1.551 *p* = .2129
	Estuarine worm	10.2	10.9	*χ* ^2^ = 0.049 *p* = .8248
	Mussel	36.6	33.1	*χ* ^2^ = 0.614 *p* = .4332
	Oyster	2.4	2	*χ* ^2^ = 0.093 *p* = .7604
	*S. plana*	8.3	6.9	*χ* ^2^ = 0.334 *p* = .5632
	Winkle	5.9	4.8	*χ* ^2^ = 0.2304 *p* = .6312
Powderham Sands	Cockle	16.7	13.2	*χ* ^2^ = 0.1228 *p* = .726
	Estuarine Worm***	0	47.4	*χ* ^2^ = 12.565 *p* = .0004
	*S. plana***	83.3	39.5	*χ* ^2^ = 9.447 *p* = .002
Exmouth RFC & FC	Earthworm	100	94.1	*χ* ^2^ = 1.893 *p* = .1689
	Leatherjacket	0	5.9	*χ* ^2^ = 1.893 *p* = .1689
Exton	Cockle	4.8	3.8	*χ* ^2^ = 0.083 *p* = .7733
	Estuarine Worm**	0	14.1	*χ* ^2^ = 9.489 *p* = .002
	Mussel	6.5	5.1	*χ* ^2^ = 0.112 *p* = .738
	*S. plana*	64.5	57.7	*χ* ^2^ = 0.674 *p* = .4115
	Winkle	24.2	19.2	*χ* ^2^ = 0.505 *p* = .4772

**p* < .05, ***p* < .01, ****p* < .001.

## APPENDIX 5

### Prey calorie content calculations

At each site where prey samples were collected and foraging behaviors were observed (Figure 5a,b,d), a prey energy value was approximated for the most commonly foraged prey (i.e., earthworms *Lumbricidae* sp. at all field sites, and at estuary sites 1 and 2 (Cockle Steps and Cockle Sands) cockles (*Cerastoderma edule*), at estuary site 3 (Lympstone) mussels (*Mytilis edulis*), at estuary site 4 (Exton) *Scrobicularia plana*). The most commonly observed prey item foraged at Powderham were estuarine worms (*Neresis* sp.), but none were collected in prey samples. Since the average mass of estuarine worms in the Exe Estuary could not be determined, the calorific value of the second most commonly foraged prey at Powderham (*S. plana*) was used at this location.

To approximate the prey energy content, the mean wet mass (including the shell if present) of each prey item was recorded in all quadrat samples. For earthworms, the estimated calorie content per earthworm was calculated as the average dry weight mass of an earthworm (0.07 g) multiplied by 3.778 kcal g^−1^, yielding approximately 0.265 kcal per earthworm (Heppleston, 1971). Then, the number of earthworms consumed during a 5‐min foraging observation was multiplied by 0.265 kcal to generate an estimate of the prey value of field foraging sites. For estuarine prey, the wet mass of cockles, mussels, and *S. plana* was converted to ash‐free dry weight (AFDW) using a conversion factor from Ricciardi and Bourget (1998). The energy content (kJ g^−1^ AFDW) of an average‐sized cockle, mussel, and *S.plana* in the Exe Estuary were then estimated using values reported by Zwarts and Wanink (1993) of 21.7, 23.3, 21.2 kJ g^−1^, respectively, and converted to kcal g^−1^ by dividing by 4.184. The calculations resulted in an estimated kcal per prey item (0.96 kcal, 2.22 kcal, and 0.064 kcal for cockles, mussels, and *S. plana*, respectively). Finally, the number of prey consumed during a 5‐min foraging observation was multiplied by the calorie value of the most commonly consumed prey species at that site to generate an estimate of the prey value of estuarine foraging sites. For cockles and *S. plana*, some quadrat samples included prey that were smaller than those likely eaten by oystercatchers, so prey with a mass in the lowest 0.25 percentile were excluded.

**Table:**

Prey species	Sample size	Mean wet mass (g) ± SE
Cockle	580 (all) 434 (>0.25 percentile)	2.47 ± 0.1 (all) 3.199 ± 0.11 (>0.25 percentile)
Mussel	5	6.86 ± 0.92
Peppery furrow shell *Scrobicularia plana*	282 (all) 199 (>0.25 percentile)	0.168 ± 0.012 (all) 0.219 ± 0.016 (>0.25 percentile)

## APPENDIX 6

The observed temperature and wind speeds during the study period that was collected at the weather station nearest to the study site. Histograms show the minimum daily temperature (a) and maximum daily wind speed (b) recorded from 14 August to 1 March. Temperatures below 0°C are indicated with a faded color and by the dashed grey line, and the Beaufort wind force scale categories are indicated by the color gradient, with high‐speed categories labeled.

## APPENDIX 7

The combined 50%, 75%, and 90% autocorrelated kernel densities calculated for each age class (A – adults, B – sub‐adults, C – juveniles).

## APPENDIX 8

The summer (April – August) and winter (September – March) locations of nonbreeding oystercatchers that remained in the Exe Estuary throughout the year.

## APPENDIX 9

### Individual variation of captured oystercatchers

The mean mass of all oystercatchers caught from February 2018 to October 2020 (*n* = 322) was greatest in adult birds (563.8 g ± 62.4 SD), followed by sub‐adults (529.1 g ± 44.7 SD) and smallest for juveniles (492.5 g ± 52.2 SD, with the lowest variation in sub‐adults; Figure 3a). Adults were significantly heavier than sub‐adults or juveniles (one‐way ANOVA F_(2, 319)_ = 15.71, *p* < .001, Tukey's HSD test was significantly different between adults and sub‐adults (*p* < .001) and adults and juveniles (*p* = .002), but not sub‐adults and juveniles (*p* = .204)). There was, however, little difference between the three age classes in the mean and variation of head and bill length (adult = 119.8 mm ± 5.5 SD; sub‐adult = 119.8 mm ± 6.1 SD; juvenile = 116.7 mm ± 10.2; one‐way ANOVA: *F*_(2,319)_ = 1.127, *p* = 0.325; Figure 3b). Adults had significantly longer wings than sub‐adults (one‐way ANOVA *F*_(2,258)_ = 9.918, *p* < .001, Tukey's HSD *p* < .001), but differences in wing length were not significant between adults and juveniles (Tukey's HSD *p* = .202) or sub‐adults and juveniles (Tukey's HSD *p* = .998; adult = 265.0 mm ± 7.2 SD; sub‐adult = 259.1 mm ± 9.9 SD; juvenile = 260.8 mm ± 7.9 SD; Figure 3c). The 24 oystercatchers (*n* = 8 adults, 11 sub‐adults, and 5 juveniles) deployed with GPS tracking devices had a mean mass of 515 g (range 407 to 583 g), a mean head and bill length of 118 mm (range 95.7–129 mm), and a mean wing length of 265 mm (range 249–294 mm [11 were not measured as they lacked wing points]). At the time of capture, the majority (54.2%) of the tracked oystercatchers had a chisel‐shaped bill (*n* = 13), 25% had a blunt‐shaped bill (*n* = 6), 16.7% had a rounded bill shape (*n* = 4), and one bill shape was not recorded. The bill types of the 24 tracked oystercatchers were representative of the proportion of each bill type of all captured individuals (*n* = 322) in the Exe Estuary from February 2018 to October 2020 (58%, 29%, and 9%, respectively, with no bill shape recorded for the remaining).

## APPENDIX 10

The 75% autocorrelated kernel density estimations (AKDE) for a subset (four of twelve) oystercatchers with location data collected for two or more winters.
